# Poly(butylene succinate-*co*-terephthalate) nanofibrous membrane composited with cyclodextrin polymer for superhydrophilic property

**DOI:** 10.1039/c7ra12068k

**Published:** 2018-01-03

**Authors:** Zhenzhen Wei, Zhijuan Pan, Faxue Li, Jianyong Yu

**Affiliations:** College of Textile and Clothing Engineering, Soochow University Suzhou 215123 China zhjpan@suda.edu.cn; National Engineering Laboratory for Modern Silk, Soochow University Suzhou 215021 China; Innovation Center for Textile Science and Technology, Donghua University Shanghai 201620 China

## Abstract

Tailoring the wetting properties of nanofibrous membranes and endowing them with expected wettability provides new ways in extending the application field of these materials. In this study, we first performed the *in situ* fabrication of poly(butylenes succinate-*co*-terephthalate) (PBST) composite nanofibrous membrane with cyclodextrin polymer (CDP) using a combination of electrospinning and heating processes. Then, the morphologies, crystallization and mechanical properties of the PBST composite membrane were investigated. It was found that the CDP was uniformly dispersed on the PBST nanofibers instead of merely covering the surface of the membrane. Moreover, the introduction of additives brought about a decreased crystallinity and tensile strength of the resultant membrane due to its restraining role in the crystallization of PBST. Furthermore, the wettability of the PBST composite membranes with various amounts of additives was explored and the evolution of water spread on top of the membranes was also recorded. The membrane became superhydrophilic from hydrophobic upon increasing the amount of additives and the water droplet could completely spread within 0.2 s, which was attributed to the enlarged roughness and increased contact area of CDP on the nanofibers. A comparison between the two fabrication methods used for PBST composite nanofibrous membranes is also presented and studies on the preparation and wetting properties may shed light on polymer composite membranes that exhibit potential application in more fields.

## Abstract 2

We present a facile fabrication method for a PBST composite nanofibrous membrane with cyclodextrin polymer *via* electrospinning and heating processes. The membrane is superhydrophilic and water can completely spread on the surface within just 0.2 s.

## Graphical abstract

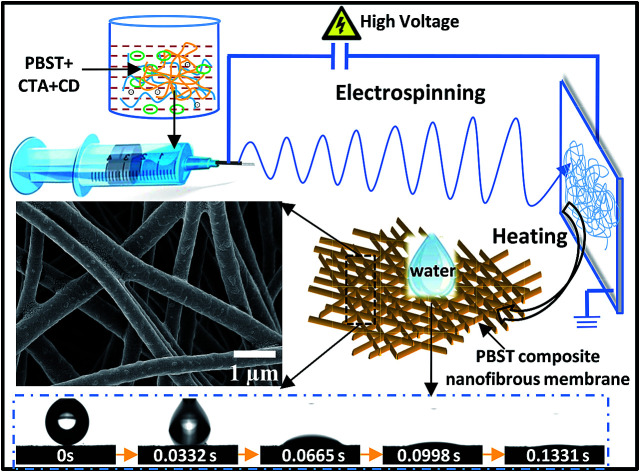

## Introduction

Aliphatic–aromatic copolymers have spurred great interest in recent years for the desirable biodegradability of the aliphatic unit and good mechanical properties of the aromatic unit in their structure.^1–3^ Poly(butylenes succinate-*co*-terephthalate) (PBST), one such copolymer, has been intensively investigated for its preparation, molecular structure and thermal and mechanical properties for applications in melt-spinning fibers and yarns.^4–9^ However, the further development of the conventional spinning method used for PBST fibers is hindered by their relatively slow crystallization rate and unstable morphologies during the manufacturing process, which in turn suppress the productivity and increase their cost.^4,7,9^ Fortunately, the rapid advance of the electrospinning technique provides hope and potential for the re-development of PBST. The preparation of PBST nanofibrous membrane *via* electrospinning has been reported, in which one of the research focuses was the effects of the spinning parameters on the structure and properties of PBST nanofibers.^10^ Moreover, it was found that the prepared PBST nanofibrous membrane exhibits a hierarchical structure with nanoscaled fibers and microscaled pores, which endows a nanofibrous membrane with hydrophobic property (water contact angle of ∼130°).^10,11^

Hydrophobic membranes, especially superhydrophobic materials, generally possess special abilities including antipollution, waterproof and self-cleaning, and show prospects in various fields such as water/oil separation.^12–15^ However, when materials are involved in the application of adsorbing small molecules in water, it is expected that the membrane can exhibit desirable hydrophilicity. It has been reported that a PBST nanofibrous membrane has been successfully used in adsorbing dyes in wastewater after immersing the membrane into a cyclodextrin aqueous solution with citric acid and heating the solution, which would form a cyclodextrin polymer (CDP) on the surface of the PBST membrane.^11^ Moreover, the results showed that although the adsorption ability was attributed to the capability of cyclodextrin to form inclusion complexes and the hydrophilicity of water-insoluble CDP,^11^ the adsorption performance was greatly limited by the preparation method used, which had the result of CDP covering most of the surface of membrane and thus it did not integrate the strength of the CDP and the nanofibers.

As a matter of fact, a large number of researchers have been devoted to investigating the modification of nanofibrous membranes using cyclodextrin or CDP *via* solution mixing or surface modification, with the aim of functionalizing these membranes and extending their application fields.^16–21^ However, their research emphases are mainly on how to fabricate water-insoluble cyclodextrin, *i.e.* CDP,^18,20,21^ and on the adsorption or separation properties of composite membranes,^17–21^ while the wettability of the nanofibrous membrane with CDP has not deeply studied, which may have great influence on their performance.

In this contribution, we present the fabrication of PBST composite nanofibers with CDP using a combination of electrospinning and heating processes. The cyclodextrin and citric acid were both added into the electrospinning solution and the PBST/CDP composite nanofibrous membranes were successfully achieved upon heating. Then, the composite nanofibrous membranes were investigated in terms of their morphology, crystallization and mechanical properties, and the wettability of the PBST nanofibrous membrane with different amounts of cyclodextrin added was also studied.

## Experimental

### Materials

**Fig. 1:**
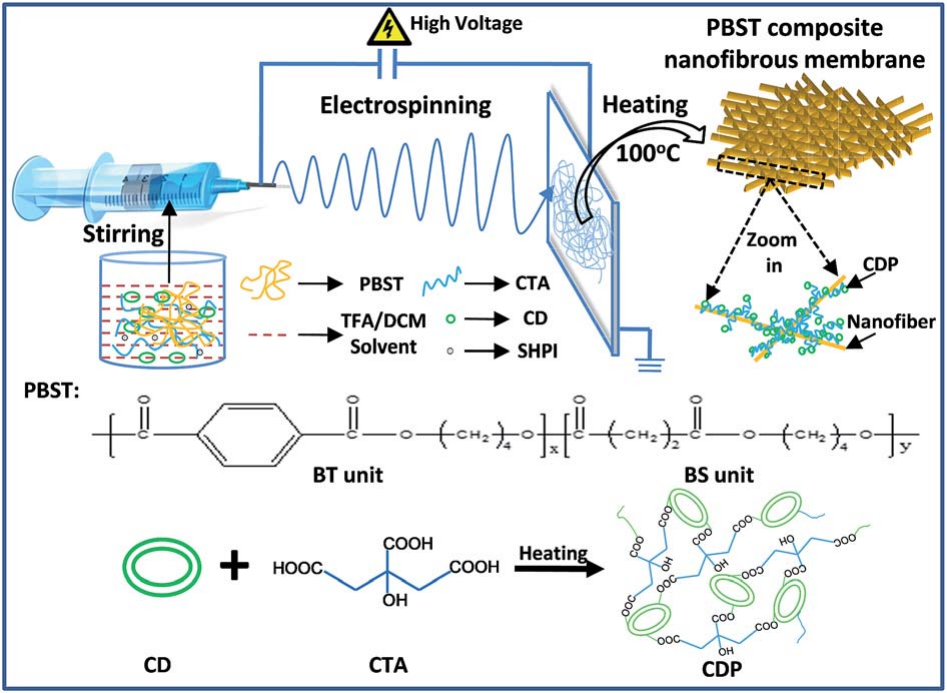
Schematic representation of the preparation of PBST composite nanofibrous membrane.

PBST pellets (*M*_n_ = 50 600 g mol^−1^, polydispersity index = 1.92) were provided by Jiangsu Heshili New Materials Co., Ltd, China and its chemical structure is shown in Fig. 1. 70 mol% of the butylenes terephthalate (BT) unit was used for the balance between desirable biodegradability and good mechanical properties.^6^ Trifluoroacetic acid (TFA) and dichloromethane (DCM) were supplied by Aladdin Chemical Regents Co., Ltd., China. β-cyclodextrin (CD), citric acid (CTA) and sodium hypophosphite hydrate (SHPI) were purchased from Sinopharm Chemical Reagent Co., Ltd. All chemicals were of analytical grade and used as received without further purification.

### Preparation of the PBST composite nanofibrous membranes

A pure PBST nanofibrous membrane and the composite membranes were fabricated *via* electrospinning and heating processes, as shown in Fig. 1. Typically, a 25 wt% PBST solution was initially prepared by dissolving PBST pellets into a mixed solvent of TFA and DCM at a volume ratio of 1 : 1 with vigorously stirring for 2 h. Then, a certain amount of CD, CTA and SHPI (as a catalyst) at a mass ratio of 1 : 1 : 0.1 were added into the above solution and stirred vigorously for another 2 h. The resulting solution was loaded into a syringe with a metallic needle to perform the electrospinning process. A high voltage of 20 kV and feed rate of 1 mL h^−1^ were applied with the distance between the needle tip and collector being 13 cm. The relevant temperature and humidity during electrospinning step were kept at 20 ± 2 °C and 45 ± 2%, respectively. Then, the nanofibrous membranes were dried in an oven at 100 °C for 2 h. As a control group, only CD, CTA and SHPI were added into the mixed solvent of TFA and DCM with vigorous stirring for 2 h and then the solution was heated at 100 °C for 2 h. For brevity, the control group was referred to as CDP and the PBST with 0, 3.7, 6.6, 9.4, 18.7 and 28.1 wt% CD were abbreviated as PBST, P–C-3.7%, P–C-6.6%, P–C-9.4%, P–C-18.7% and P–C-28.1%, respectively.

### Characterization

The morphologies of the PBST composite nanofibrous membranes were examined using field emission scanning electron microscopy (FE-SEM) with a Hitachi S-4800 (Japan) at an accelerating voltage of 3.0 kV. The nanofibers were coated with gold prior to the SEM examination. The surface area of the composite membranes was determined using Brunauer–Emmett–Teller (BET) analysis on a Micromeritics ASAP-2020 analyzer. The pore size of each nanofibrous membrane was measured using a Porometer 3G through-pore size analyzer.

The Fourier transform infrared spectra (FTIR) of the PBST composite nanofibrous membranes were recorded using a FTIR spectrometer (Nicolet 6700, Thermo Fisher). The PBST composite nanofibrous membranes were also analyzed on a differential scanning calorimeter (Pyris-1 DSC, PerkineElmer). Around 4 mg of sample sealed in an aluminium pan was heated to 210 °C at a rate of 10 °C min^−1^ and kept at this temperature for 3 min before cooling at a rate of 10 °C min^−1^ to 30 °C under an N_2_ atmosphere. For the solubility experiments, 5 cm^2^ of the fiber mats were immersed in deionized water at room temperature. After 24 h, the fiber mats were removed from the deionized water and placed in a vacuum oven to dry until constant weight. The insoluble fraction (%) was determined using the ratio of weight of sample after drying in an oven to the weight of the initial sample. Tensile tests of the specimens with dimensions of 50 mm × 5 mm were conducted on an Instron Universal Testing Machine. A 20 mm gauge length and a cross-head speed of 20 mm min^−1^ were used during each test at room temperature. The values were averaged over 5 trials.

The water contact angles of the nanofibrous membranes were measured using an optical contact angle measuring system (Krüss DSA100, Germany) and the changing contact angles after a 3 μL droplet was placed on the membranes were also recorded.

## Results and discussion

**Fig. 2:**
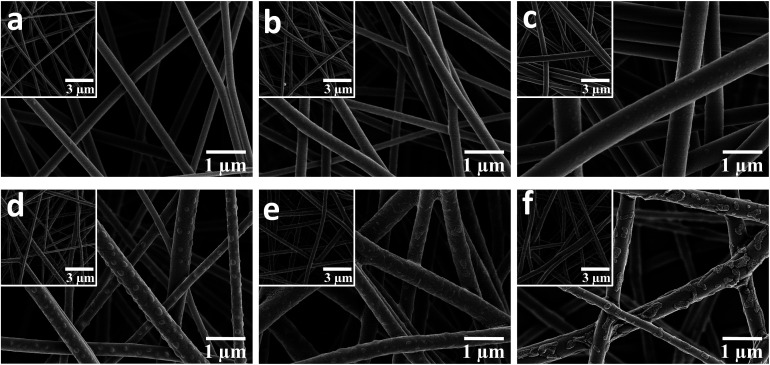
SEM images of the nanofibrous membranes: (a) pure PBST, (b) P–C-3.7%, (c) P–C-6.6%, (d) P–C-9.4%, (e) P–C-18.7% and (f) P–C-28.1% (the insets are the corresponding images at lower magnification).

The representative SEM images of the pure PBST nanofibrous membranes shown in Fig. 2a illustrate that the pure PBSTnanofibers were oriented randomly with an average diameter of 269 nm (listed in Table 1) and also exhibited a very smooth surface. When the PBST composite membranes were fabricated (Fig. 1b–f), embossments could be observed on the surface of the nanofibers, which gave rise to a larger average diameter and standard deviations (Table 1) than those found for pure PBST. Meanwhile, it was observed that these separated embossments (Fig. 1c and d) changed into adhesive pieces (Fig. 1e and f) upon increasing the amount CD added. According to the preparation steps of the PBST composite membranes and relevant references,^11,17^ the embossments on the surface of the nanofibers were speculated to be cyclodextrin polymer (CDP), which will be justified later. Furthermore, as summarized in Table 1, the surface area of the composite membranes increased from 18.5 to 26.8 m^2^ g^−1^ after adding the CD and CTA into the electrospinning solution, whereas the pore size showed a decreasing trend due to the embossments formed on the nanofibers. The results of surface area and pore size variation were not consistent with other relative reports,^17,22^ in which smooth nanofiber surfaces were observed in all the composite nanofibers reported. The differences in the nanofibers morphology between our membrane and the previously reported composite membranes can be attributed to the different polarity of the polymers. PBST is a non-polar polymer without any functional groups, while in the literature, the polymers (PVA and PAA) they used for electrospinning are polar with carboxyl groups, which have a tendency to cross-link with cyclodextrin during the heating process with a result of integrating together with a smooth surface.

**Table 1:** Average diameter, surface area and pore size of the PBST composite nanofibrous membranes

Sample	PBST	P–C-3.7%	P–C-6.6%	P–C-9.4%	P–C-18.7%	P–C-28.1%
Diameter (nm)	269 ± 36	302 ± 43	564 ± 44	309 ± 95	417 ± 81	396 ± 93
Surface area (m^2^ g^−1^)	18.5	20.2	24.6	25.9	27.5	26.8
Pore size (μm)	3.41 ± 0.26	3.25 ± 0.41	2.74 ± 0.46	2.47 ± 0.22	1.98 ± 0.19	1.91 ± 0.17

**Fig. 3:**
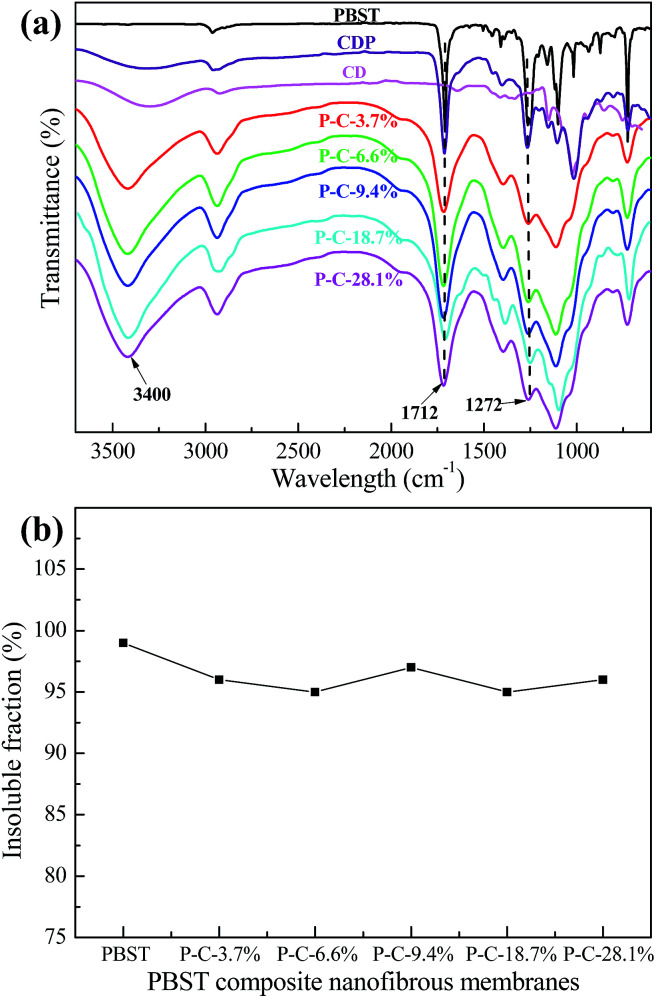
(a) Infrared spectra of CD, CDP and the PBST composite nanofibrous membranes, and (b) the insoluble fraction of the PBST composite membranes after soaking in water for 24 h.

In order to verify the above speculation that the embossments can be assigned to CDP, the infrared spectra of CD, CDP and the PBST composite nanofibrous membranes were recorded and presented in Fig. 3a. As shown in this Fig. 3a, when compared to CD, there are two evident adsorption bands (1712 and 1272 cm^−1^) in the spectrum of CDP, which should be assigned to the C=O stretching vibration and C–O stretching vibration of the ester groups, respectively.^22^ These newly emerging bands indicate that the hydroxyl groups of CD reacted with the carboxyl groups of CTA to form ester groups, suggesting that the cross-linking reaction between CD and CTA had occurred.^23,24^ Here, we have to point out that CDP was formed in the process of heating the as-prepared membranes at 100 °C for 2 h rather than in the electrospinning process. Under heating, the hydroxyl groups of β-cyclodextrin (CD) can react with the carboxyl groups of citric acid (CTA) to form ester groups. Meanwhile, in comparison with pure PBST, the spectra of CDP and the PBST composite membrane all exhibit a broad adsorption band in wavelength of ∼3400 cm^−1^, which should correspond to the –OH stretching vibration in CD and CDP.^22^ The broad peak also suggested that there does exist the newly emerging material CDP in the PBST composite nanofibrous membranes. Furthermore, the spectra of the PBST composite membranes in Fig. 3 show no newly formed adsorption bands when compared with CDP and pure PBST, suggesting that no chemical reaction occurred between CD/CTA and PBST nanofibers due to the lack of free reactive groups in the PBST macromolecules.

Fig. 3b shows the insoluble fraction of the PBST composite nanofibrous membranes after soaking in water for 24 h at room temperature. The insoluble fraction of the PBST composite nanofibrous membranes after soaking in water for 24 h were all larger than 95%, indicating these composite membranes had a good resistance to water, *i.e.* high water-insoluble level, and the CDP was also water-insoluble. This could be attributed to the extra hydroxyl groups and carboxyl groups in CD, CTA and the intermediate products, the esterification and polymerization reactions upon heating and finally, the CDP with a three-dimensional network structure being formed, which in turn could make the nanofibrous membrane useful for aqueous medium applications.

**Fig. 4:**
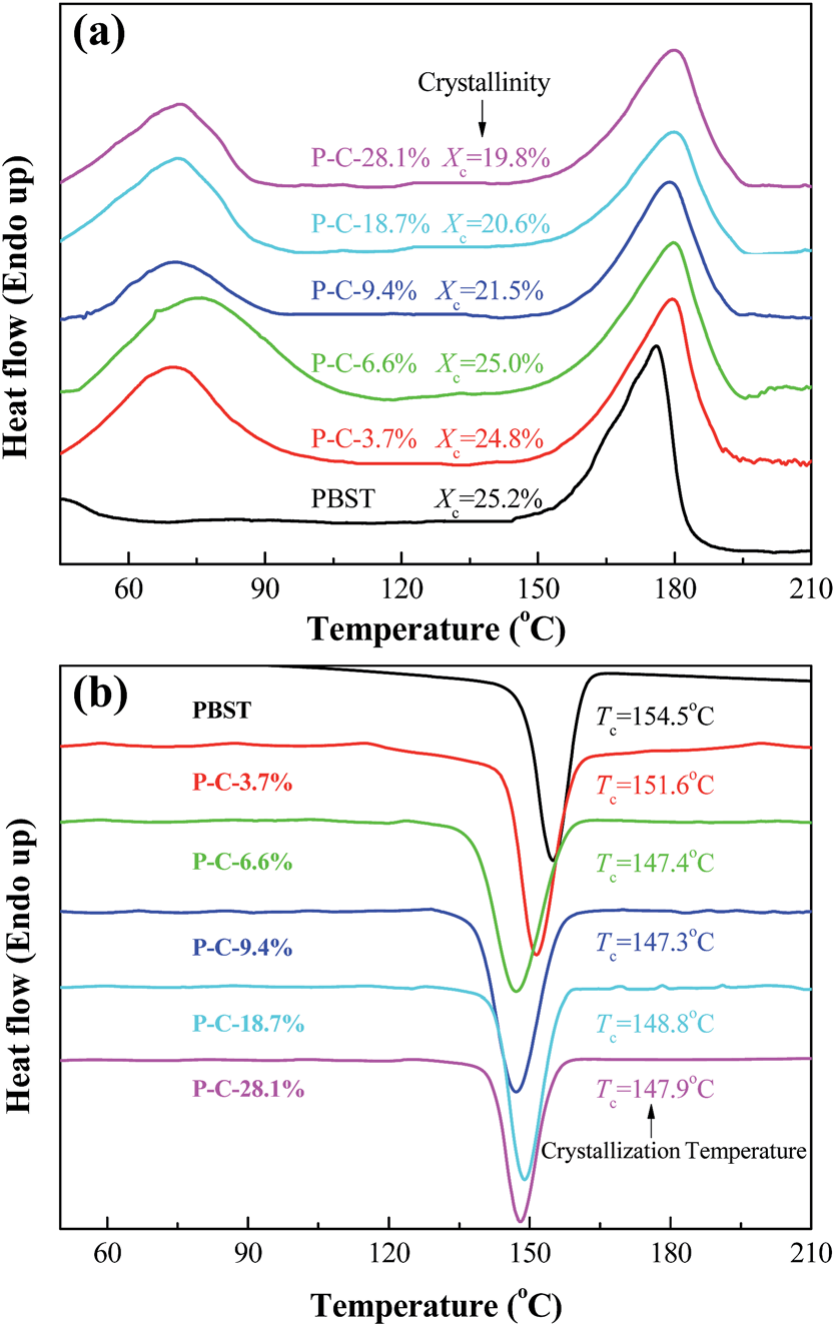
Heating scan (a) and cooling scan (b) in the DSC curves obtained for the PBST composite nanofibrous membranes.

DSC was used to investigate the effects of the CD and CTA added to the electrospinning solution on the crystallization behavior of PBST, as shown in Fig. 4. Upon increasing the amount of CD and CTA added, the crystallinity (*X*_c_) of PBST decreased. Here, the crystallinity is the ratio of the melting enthalpy in heating curve (the second peak in Fig. 4a) to the melting enthalpy of sample with 100% crystallinity (142 J g^−1^).^25^ The reduced crystallinity of PBST can be attributed to the presence of CD and CTA during the electrospinning step and the formation of CDP during the subsequent heating step, which to some extent lowered the regularity of the molecular chains of PBST, which were arranged in ordered ways during the process of spinning and hindered the recrystallization in the heating process. In comparison with pure PBST, a new endothermal peak was observed for the PBST composite membranes (a broad peak from 60 to 100 °C), which was due to the evaporation of water stored in the CDP in the PBST composite nanofibers.^11^

Fig. 4b presents the cooling curves of the PBST composite nanofibrous membranes after heating. Only one crystallization peak was observed in the cooling process, which can be assigned to the exothermal crystallization of PBST. It has been reported that CDP possesses a three-dimensional network of cross-linking macromolecules, which will not crystallize upon cooling after melting.^11^ Meanwhile, the crystallization temperature (*T*_c_) of PBST was reduced upon increasing the amount of CD and CTA added. In the electrospinning process, the molecular chains of PBST are arranged in an ordered way *i.e.* crystallization with the evaporation of solvent and at the same time, the molecules of CD and CTA in solution are be repelled to the outside of the PBST, resulting in hindering this ordered arrangement. The more CD and CTA added, the less likely the crystallization of PBST was to occur.

**Fig. 5:**
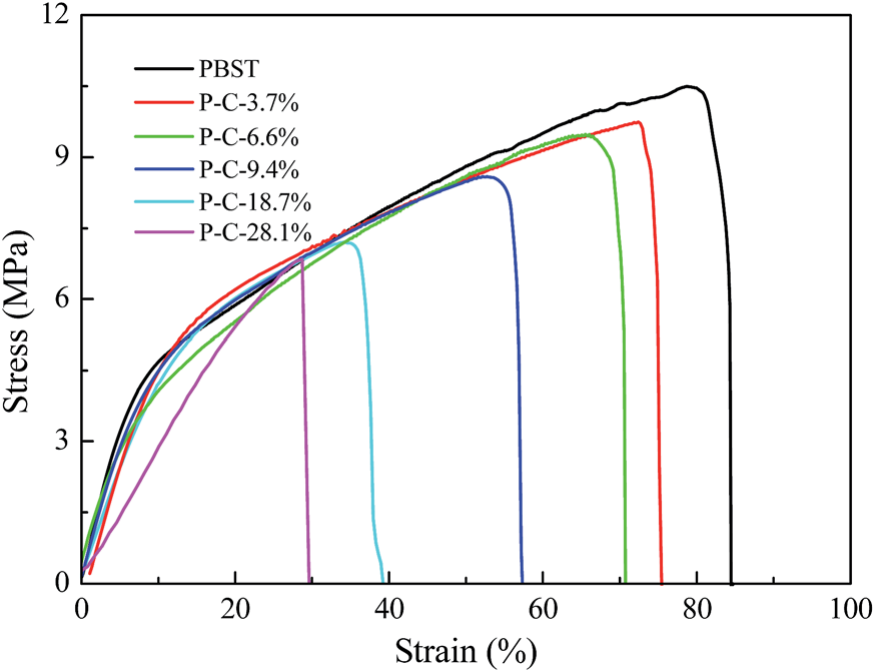
Stretching curves obtained for the PBST composite nanofibrous membranes.

The morphologies and crystallization behaviour discussed above must influence the mechanical properties of the PBST composite nanofibrous membranes and Fig. 5 shows their stretching stress strain curves. Among all the nanofibrous membranes, pure PBST exhibited the largest tensile strength and elongation at break. When the CD and CTA were introduced and formed CDP on the surface of PBST nanofibers, the strength and elongation of the composite membranes decreased with an increase in the amount of CD and CTA added. The decline in the mechanical properties may be attributed to the following aspects: (1) the decreased crystallinity inevitably results in reduced strength; (2) the CDP is separately dispersed between the nanofibers and physically adhered to the surface of the nanofibers, which leads to the reduced cohesive force between the fibers and weakens the mechanical properties;^26^ (3) different from other reported polymers (PVA and PAA) composited with CDP,^17,22^ PBST does not possess any polar groups, such as hydroxyl or carboxyl, and cannot cross-link with CD and/or CTA, resulting in weakened mechanical properties.^17,22^

**Fig. 6:**
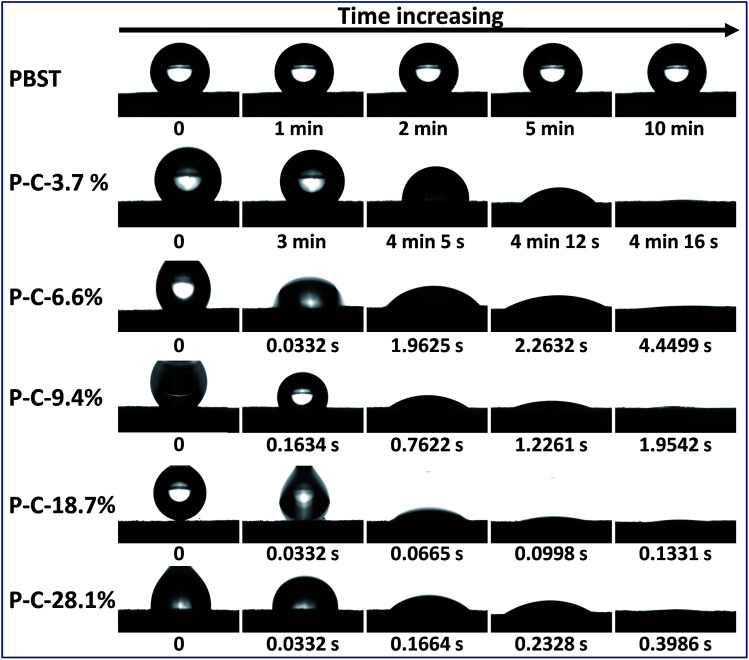
Evolution of a water droplet placed on the PBST composite nanofibrous membranes with time.

It has been reported that CD is water-soluble, CDP is water-insoluble and both have an outstanding capability to form inclusion complexes with a variety of molecules including water molecules, *i.e.* hydrophilic, while the pure PBST nanofibrous membrane exhibits hydrophobic properties with a water contact angle of ∼130°.^10,11^ As a result, it was necessary and of interest to study the wettability of the composite membranes when CDP and PBST were blended together by solution mixing of the monomers and thermal cross-linking. Fig. 6 shows the evolution of a water droplet placed on the PBST composite nanofibrous membranes with time. The pure PBST nanofibrous membrane was observed to be hydrophobic with a water contact angel of 136° and the water droplet kept its shape on the membrane for a very long time (10 min). When a small amount of CD and CTA was added (P–C-3.7%), the water remained unchanged within the first 3 minutes and then slowly spread along the membrane to be completely spread (water contact angle of 0°) after 4 min 16 s. This was possibly because the P–C-3.7% membrane was still hydrophobic at first and the water was absorbed by the CDP adhered to the nanofibers after a few minutes. Water started to spread once dropped on the P–C-6.6% membrane, suggesting that this PBST composite membrane had been changed into a hydrophilic surface. Upon further increasing the amount of CD and CTA added, the water spread on the composite membranes was significantly speeded up. It was noteworthy that the P–C-18.7% membrane became highly hydrophilic and the water droplet was completely spread within just 0.2 s, while when the amount reached to 28.1%, the time needed for the water contact angle to change to 0° was longer than observed on P–C-18.7%, which could possibly because the CDP was unevenly stuck in clumps on the surface of the nanofibers when the amount of CD and CTA added was too large.

**Fig. 7:**
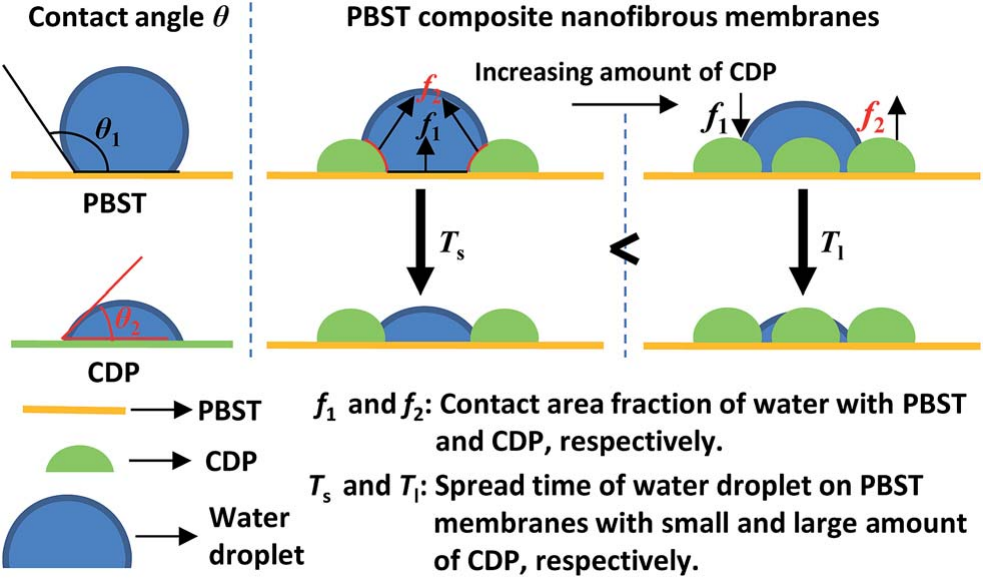
Illustration showing the evolution of the water contact angle of the PBST composite nanofibrous membranes.

According to the above results, it can be concluded that the hydrophilic properties of the PBST composite nanofibrous membranes were greatly enhanced and the corresponding reasons for this are illustrated in Fig. 7. Firstly, CDP on the surface of the nanofibers are hydrophilic;^11^ when water is coincidentally placed on the surface of CDP, the composite membrane will bet hydrophilic. Secondly, the formation of CDP and its distribution on the nanofibers increases the surface roughness of the PBST nanofibrous membrane (also shown in the SEM images); it is well-known that surface roughness makes the apparent contact angle lower when the intrinsic contact angle is less than 90°.^27,28^ So the superhydrophilicity of the composite membrane with more than 6.6% of CD and CTA added in the spinning solution also arose from the amplification effect of the surface roughness on the wettability. Furthermore, the Cassie model provides an equation (cos *θ* = *f*_1_ cos *θ*_1_ + *f*_2_ cos *θ*_2_ = (1 − *f*_2_)cos *θ*_1_ + *f*_2_ cos *θ*_2_ = cos *θ*_1_ + *f*_2_(cos *θ*_2_ − cos *θ*_1_)) used to calculate the apparent contact angle *θ* when a droplet is placed on the top of an heterogeneous solid surface,^29,30^ in which in our case *θ*_1_ and *θ*_2_ are the contact angle of water dropped on pure PBST and CDP, respectively; *f*_1_ and *f*_2_ are the contact area fraction of water in contact with PBST and CDP, respectively, and *f*_1_ + *f*_2_ = 1. Upon increasing the amount of CDP on the surface of the PBST composite nanofibers, the area fraction *f*_2_ increased, cos *θ* was enlarged and the apparent contact angle *θ* became smaller, leading to a superhydrophilic surface with a faster spread speed (*T*_s_ < *T*_l_ as shown in Fig. 7).

To date, two kinds of methods have been reported to prepare PBST composite nanofibrous membranes with CDP. One is to immerse the PBST membrane into a CD and CTA aqueous solution followed by heating the solution.^11^ The other is reported in this study, adding CD and CTA into the mixed solvent containing PBST, electrospinning the solution to form membrane and then heating the membrane. A comparison between the two methods is listed as follows: they share some common features, both methods can successfully fabricate the PBST/CDP composite nanofibrous membranes with the same theory of forming CDP, while the differences cannot be ignored. Firstly, the second method reported in this study can bring about a uniform distribution of CDP on the surface of the PBST nanofibers rather than unevenly covering the surface of the PBST membrane observed when using the first method. Secondly, the first method does bring about great changes in the structure and properties of PBST itself, while the wetting properties of the PBST composite membrane fabricated using the second method is greatly changed to superhydrophilic from hydrophobic due to the decreased regularity of the molecular chains, the formation of hydrophilic CDP and the increased roughness of the nanofibers.

## Conclusions

In summary, PBST composite nanofibrous membranes were successfully prepared *via in situ* electrospinning of a PBST solution mixed with cyclodextrin and citric acid followed by heating the membrane to form cyclodextrin polymer (CDP). The results show that CDP was evenly dispersed on the surface of the PBST nanofibers and endowed the composite membrane with increased surface area, roughness and hydrophilic material on surface (*i.e.* CDP). Meanwhile, although the introduction of cyclodextrin and citric acid into electrospinning solution has a negative influence on the crystallinity and mechanical properties of resultant membrane, the wettability of the PBST composite membrane was changed to superhydrophilicity from hydrophobicity, which can be explained by the presence of hydrophilic CDP and the enlarged roughness. Our investigation concentrated on the preparation methods and wetting properties of the nanofibrous membrane, which is beneficial for the creation of materials with desirable properties for various applications.

## Conflicts of interest

There are no conflicts to declare.

## Supplementary Material
